# Barriers for the Implementation of Liquid Biofuels

**DOI:** 10.3390/molecules26237157

**Published:** 2021-11-26

**Authors:** Juan Carlos Serrano-Ruiz

**Affiliations:** Materials and Sustainability Group, Department of Engineering, Universidad Loyola Andalucía, 41704 Seville, Spain; jcserrano@uloyola.es; Tel.: +34-955-641-600 (ext. 2579)

Transportation accounts for nearly one third of the total energy consumed worldwide and, unlike other sectors, it relies almost exclusively (96%) on petroleum. From a technical point of view, this high reliance on petroleum is perfectly understandable, since this resource provides compounds (i.e., hydrocarbons) with a number of properties (e.g., high energy density, high chemical stability, low viscosity, and clean burning) that make them excellent fuels. However, the mobility of goods and people, indispensable for the healthy economic development of any society, necessitates an important environmental price. Thus, the vigorous and fast-growing transportation sector is responsible for approximately one fifth of the greenhouse gas emissions released to the atmosphere every year [1]. The reduction of these emissions inevitably leads to the utilization of liquid fuels from sources other than petroleum, with biomass being the only renewable source of carbon available today. However, this transition from petroleum to biomass, currently being pushed by many governments worldwide, is not straightforward, and two obstacles are particularly important. First, there is a technological barrier that ultimately originates from the very different nature and chemical composition of biomass and petroleum. Thus, many of the technologies developed and optimized over the past decades for petroleum are not suitable to process biomass feeds, making it necessary to design new routes and to build new knowledge on the behavior of these new highly reactive and complex biomass feeds (e.g., sugars, platform molecules, lipids, and lignocelluloses). Ideally, these new biofuel technologies should be able to compete in efficiency (at least in the medium and long terms) with their highly optimized petroleum counterparts while providing compounds with fuel characteristics close to those of hydrocarbons. Second, there is an infrastructure barrier that hinders (and in some cases avoids) the penetration of new biofuels in the transportation market. The entire transportation infrastructure (e.g., engines, fueling stations, distribution networks, and storage tanks) has been developed to accommodate petroleum hydrocarbons. Again, ideally, the new liquid biofuels should be designed in a way they can adapt to this hydrocarbon-based infrastructure, avoiding very disruptive approaches.

While the ideal situation exposed above is far from being achieved, there are reasons to be optimistic. The share of liquid biofuels in the transportation sector has increased steadily in the last 10 years, from 59 million tonnes of oil equivalent (Mtoe) in 2010 to 96 Mtoe in 2019 [2], and this trend is expected to continue in the coming years. Two biofuels have been successfully developed, contributing to this growing trend: biodiesel (i.e., long-chain alkyl esters produced by transesterification of triglycerides), and, especially, bioethanol (produced by bacterial fermentation of sugars). These oxygenated biofuels have overcome the technology barrier by making use of simple chemistry and well-known biological processes (i.e., transesterification of triglycerides and bacterial fermentation of sugars). The penetration of these biofuels in the transportation market, while being significant, is not sufficient to reach the targets of the 2030 agenda for sustainable development. This can be explained because these biofuels have overcome the infrastructure barrier only to a certain extent. In the case of bioethanol (accounting for 90% of the total biofuel production), its corrosiveness, hygroscopicity, and low energy-density (23.4 vs. 34.4 MJ/L for gasoline) discourage its use as a pure fuel in spark ignition engines, and only low concentration blends with gasoline (5%–10% *v/v*, E5–E10 blends) can be assimilated by the transportation infrastructure. This “blend wall”, in fact, has prevented the U.S. (the world largest bioethanol producer) from increasing its bioethanol production above 10% of the total U.S. motor gasoline consumption in the last 10 years [3]. Alternatives involving changes in the infrastructure such as the flexible fuel vehicles (FFVs) able to run on E85 mixtures have been proposed to solve this issue, but the fact that the bioethanol production volume has been dictated over the last decade by the blend wall is a strong indicator that these measures are not easy to implement.

The above analysis indicates that, if we want to increase the share of biofuels and meet the 2030 targets, it is necessary to develop alternatives that can be more easily assimilated by the existing infrastructure (including petroleum refineries). In this sense, the conversion of biomass into liquid hydrocarbon fuels chemically identical to those used today in the transportation sector represents an interesting approach, particularly for sectors such as the aviation in which the stringent energy-density and cold-flow requirements prevent oxygenated fuels from being used [4]. The production of these green hydrocarbons, however, implies profound chemical changes and requires significant oxygen removal from biomass, ultimately leading to complex processing and multistep routes. The technological barrier is therefore the main obstacle here, and efforts should be focused on decreasing the complexity of these routes. This can be carried out, for example, by developing multifunctional catalyst able to promote several reactions in a single reactor [5] or, alternatively, by using biomass feeds with a composition closer to the final hydrocarbon fuel (e.g., vegetable oils) [6]. The production of green hydrocarbons seems necessary to provide the transportation sector with a higher number of biomass-derived alternatives so that both technological and infrastructure barriers can be overcome in the near future.
